# Towards a consensus definition of immune exclusion in cancer

**DOI:** 10.3389/fimmu.2023.1084887

**Published:** 2023-03-22

**Authors:** Ankur Tiwari, Tamas Oravecz, Laura A. Dillon, Antoine Italiano, Laurent Audoly, Wolf Hervé Fridman, Guy Travis Clifton

**Affiliations:** ^1^ Department of Surgery, University of Texas Health Science Center San Antonio, San Antonio, TX, United States; ^2^ Parthenon Therapeutics, Boston, MA, United States; ^3^ Institut Bergonié, University of Bordeaux, Bordeaux, France; ^4^ Centre de Recherche des Cordeliers, National Institute for Health and Medical Research (INSERM), Sorbonne Université, Université Sorbonne Paris-Cité (USPC), Université de Paris, Equipe Inflammation, Paris, France

**Keywords:** immune phenotypes, immune exclusion, tumor microenvironment, immune cell topography, cancer immunotherapy

## Abstract

**Background:**

The immune cell topography of solid tumors has been increasingly recognized as an important predictive factor for progression of disease and response to immunotherapy. The distribution pattern of immune cells in solid tumors is commonly classified into three categories - namely, “*Immune inflamed*”, “*Immune desert”* and *“Immune excluded” -* which, to some degree, connect immune cell presence and positioning within the tumor microenvironment to anti-tumor activity.

**Materials and methods:**

In this review, we look at the ways immune exclusion has been defined in published literature and identify opportunities to develop consistent, quantifiable definitions, which in turn, will allow better determination of the underlying mechanisms that span cancer types and, ultimately, aid in the development of treatments to target these mechanisms.

**Results:**

The definitions of tumor immune phenotypes, especially immune exclusion, have largely been conceptual. The existing literature lacks in consistency when it comes to practically defining immune exclusion, and there is no consensus on a definition. Majority of the definitions use somewhat arbitrary cut-offs in an attempt to place each tumor into a distinct phenotypic category. Tumor heterogeneity is often not accounted for, which limits the practical application of a definition.

**Conclusions:**

We have identified two key issues in existing definitions of immune exclusion, establishing clinically relevant cut-offs within the spectrum of immune cell infiltration as well as tumor heterogeneity. We propose an approach to overcome these limitations, by reporting the degree of immune cell infiltration, tying cut-offs to clinically meaningful outcome measures, maximizing the number of regions of a tumor that are analyzed and reporting the degree of heterogeneity. This will allow for a consensus practical definition for operationalizing this categorization into clinical trial and signal-seeking endpoints.

## Introduction

Since ipilimumab, the first approved checkpoint inhibitor, was FDA approved 2010 (1), cancer immunotherapy has evolved into a major therapeutic option that has revolutionized the treatment of multiple solid and hematological malignancies. The mechanism of action of checkpoint inhibitors relies largely on augmenting pre-existing anti-tumor T-cell responses (2–4). Ipilimumab, for example, promotes anti-tumor immunity by blocking the immune checkpoint cytotoxic T-lymphocyte antigen-4 (CTLA-4), which is a down-regulator of T-cell activation (5). Programmed cell death protein 1 (PD-1), another target for cancer immunotherapy, is also an inhibitory receptor on T-cells and expression of its ligand, PD-L1, by neoplastic cells, as well as by myeloid cells in the tumor microenvironment (2), is thought to be a major mechanism by which tumors evade killing by the immune system (6, 7). Anti-PD-1 drugs, such as pembrolizumab (8, 9) and nivolumab (10, 11), and anti-PDL1 antibodies such as atezolizumab (2) and durvalumab (12), act by preventing T-cell PD1/PD-L1 interaction, leading to restoration of T-cell mediated anti-tumor immunity (13).

Not all patients see clinical benefit from immunotherapy. The response rates to checkpoint inhibitors range from 10-60% to initial treatment, and many who initially respond will eventually develop secondary resistance (14, 15). Overcoming this resistance by understanding and addressing its mechanisms is key to improving the success of immunotherapy. One of the proposed mechanisms for primary resistance to immunotherapy for some patients is the inability of effector immune cells at the site of the tumor to infiltrate into the tumor parenchyma to interact with cancer cells, a phenomenon known as immune exclusion (4). This paper will discuss the concept of immune exclusion, review the ways it has been defined in the published literature, and discuss opportunities to develop a consensus definition.

## Concept of immune exclusion

**Figure 1:**
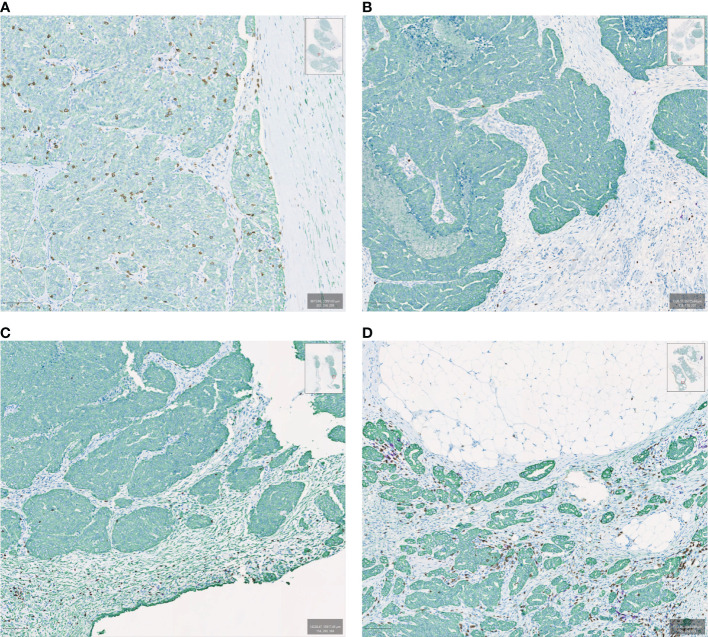
Examples of immune spatial phenotypes in ovarian cancer samples evaluated by multiplexed immunohistochemistry. Samples are stained with CK (green), CD8 (brown), and CD20 (purple). **(A)** Immune inflamed, **(B)** Immune desert, **(C)** Immune excluded with CD8 lymphocytes peripheral to the tumor bed, **(D)** Immune excluded with CD8 lymphocytes in the tumor bed but confined to the stroma.

In solid tumors, immune cell topography, which refers to the spatial distribution of immune cells in the tumor microenvironment (TME), has emerged as an important predictor of outcome as well as responsiveness to therapy (16–18). The most frequently used method for defining tumor topography is immunohistochemistry (IHC), which allows quantification of the type, density, and localization of the immune cells in relation to other cell types (19). In that regard, it is important to outline certain basic concepts of solid tumor histopathology. Broadly, solid tumors consist of the *tumor parenchyma*, containing nests of tumor cells, and the *tumor stroma*, in which the tumor cells are dispersed, containing the connective tissue, blood vessels, and often inflammatory cells (20). The *“invasive margin”* is typically defined as a 1-mm region centered on the border separating the malignant cell nests from the host tissue. The *“central tumor”* represents the remaining tumor area (21). Based on IHC analyses over the years, distinct patterns of immune cell infiltration have been identified according to the presence and type of immune cells as well as their proximity to tumor cells. As a result, the TME can be histopathologically classified into three basic descriptive immune profiles (2, 4, 22). The “*Immune-active*” or “*Immune-inflamed*” or “*hot*” phenotype is characterized by lymphocytic infiltration in the tumor parenchyma, with the immune cells positioned in proximity to the tumor cells (2, 22–24). The “*Immune-desert*” or “*cold*” phenotype is typically characterized by a lack of lymphocytes in either the tumor parenchyma or the periphery of the tumor, although in many cases it has been used to describe tumors that lack lymphocytes in the center of the tumor without regards for the periphery (2, 22–24). The *“Immune-excluded”* phenotype is a more recent distinction that is characterized by an abundance of immune cells in the TME; however, they are confined to the stroma of the tumor and do not penetrate the parenchyma of the tumors (**Figure 1**). Chen and Mellman first identified immune exclusion as a separate TME phenotype category (4) but the concept and the association with resistance to checkpoint inhibitor therapy was previously described by others (2, 22, 25).

These tumor immune phenotypes can also be defined immunologically, based on the likely rate-limiting step in the cancer-immunity cycle.(4) In this regard, the “*Immune-desert”* phenotype implies a lack of pre-existing anti-tumor immunity reflecting that the generation of tumor-specific immune cells is the rate limiting step. The *“Immune-excluded”* phenotype implies a successful generation of tumor-specific immune cells, with penetration of the tumor as the possible rate limiting step. The *“Immune-inflamed”* phenotype suggests successful generation and infiltration of immune cells, with functional suppression of the immune response in the tumor bed. Thus, the functional phenotype(s) of the immune cells that are present in infiltrated/excluded tumors is also of major significance when considering the anti-tumor immune response. It is especially relevant in the *“Immune-inflamed”* tumors, where the immune cells despite being in proximity to cancer cells are unsuccessful in clearing them, often expressing an “exhausted” or dysfunctional phenotype (26). This, however, represents a distinct mechanism of immune escape by tumors and so purely in terms of defining the immune cell topography, the phenotype(s) of the immune cells is not a focus of this review.

The biology that underlies immune exclusion is an area of active investigation, with several hypotheses proposed for potential non-mutually exclusive mechanisms. Pai et al. in 2020 (27) categorized the barriers to the immune cells as mechanical, functional, or dynamic. Mechanical barriers act as a physical impediment to a direct contact between immune cells and cancer cells. These include stromal fibrosis in the tumor periphery, with TGFβ mediated fibrotic responses and epithelial to mesenchymal transition playing a critical role (28, 29). Disordered vascularization of the tumor also likely contributes to the physical exclusion of immune cells, with endothelial receptors involved in translocation (30, 31) as well as VEGF (32) playing prominent facilitator or inhibitor roles in mediating access of immune cells into tumors. Functional barriers form an immunosuppressive milieu instigated by the tumor’s metabolic activity and interaction with stromal cells which limit migration, function, and/or survival of T-cells. These involve metabolic alterations such as the Warburg or reverse Warburg effect (33, 34), with the resultant acidic TME as well as hypoxia leading to depressed T cell function (35–37). Factors like TGFβ and VEGF, in addition to inducing physical barriers, are also involved in suppression of immune cell function. (29, 38). Another hypothesis for immune exclusion is an abruptly decreasing gradient at the periphery of chemokines such as CXCR-3 and CCR-5, that are implicated in the recruitment of T-cells (39). One phenomenon that is also postulated to contribute to functional barriers is a dampening of the inflammatory response to cellular stress and death. This involves mechanisms such as adenosine signaling, TAM receptor kinases and CD47/SIPR-α interactions (40–43). A final category of potential functional mechanisms of immune exclusion is tumor cell-intrinsic signaling that modulates chemo-attraction and immune modulatory responses. The major pathways in this category are the STAT3 (44, 45), PI3K (46), MAPK (47) and β-catenin pathways (48–50). Dynamic barriers are induced after cancer cells interact with T-cells, resulting in limited T-cell function. These include, but are not limited to, checkpoint receptor/ligand interactions occurring at the tumor periphery such as the inducible activation of PDL-1 in response to IFN-γ production by stimulated T-cells (51).

The tumor immune phenotypes have been shown to be linked with prognosis. Though the data is understandably varied, with tumor type and treatment among other factors that would need to be considered, the immune inflamed (or equivalent) phenotype is consistently associated with better outcomes. In majority of the studies, the immune excluded phenotype corresponds to intermediate prognosis and the immune desert phenotype has worst outcomes (19, 52, 53). Some studies, however, show the excluded phenotype to have even worse outcomes than the immune desert tumors (54, 55). Studies have also linked immune phenotype with response to immunotherapy, with most of them showing lack of response in the non-inflamed (excluded and desert) phenotypes (2, 14, 22, 53, 56). Thus, patients with immune excluded tumors represent a group in need of novel therapies. Targeting the unique mechanisms behind immune exclusion may serve to improve cancer outcomes across a range of cancer types, including by potentiating therapeutic benefit of existing immunotherapies in patients who would not otherwise respond. To date, there is no accepted or consensus definition for immune exclusion. In order to better study and treat these patients, it will be helpful to have an agreed upon, consensus definition of the immune excluded phenotype, an ill-defined tumor category that is often described qualitatively (4). This could allow for a more uniform approach to the identification of immune exclusion in different clinical contexts and help identify points of therapeutic intervention.

## Previously applied definitions of immune exclusion

To better understand immune exclusion, as well as to correlate tumor immune phenotype with response to immunotherapy and overall outcomes, multiple studies have categorized the tumor microenvironment using a variety of different methods to arrive at objective and practical definitions of cancer immune phenotypes. Most studies have relied on IHC, which has been the conventional method to analyze immune cell distribution. Almost all studies utilize surgically resected tissue. Although theoretically, core needle biopsies can be analyzed similarly, they provide only a small sample of the entire tumor and may not capture the relevant tumor compartments or interface. They will provide less information on tumor heterogeneity, as addressed later. In terms of the subsets of immune cells, a majority of the studies have looked at CD8+ T-lymphocytes, widely considered to play the dominant role in the effector immune response, as the primary classifier. Some of the studies have also analyzed additional cells including, but not limited to, CD4+ T cells, FoxP3+ T cells and CD163+ macrophages (**Supplementary Table**).

### Immunohistochemistry-based definitions

Galon et al. (16) analyzed immune infiltrates in large cohorts of colorectal cancer by performing IHC for CD3+, CD8+, CD45RO+ and GZMB+ cells. They determined median cutoff values of cell densities for each cell type in the center of the tumor (CT) and in the invasive margin (IM), and designated each tumor region (CT and IM) as high (Hi) or low (Lo) according to the cutoff. Hence, they were able to classify the tumors based on CT/IM cell densities as Hi/Hi, Lo/Lo, Hi/Lo or Lo/Hi. Among other results, their study showed the best disease-free survival in the CD3 Hi/Hi group, worst prognosis for the CD3 Lo/Lo group and intermediate outcomes in the CD3 Hi/Lo and CD3 Lo/Hi groups. Though the concept of tumor immune phenotypes was not prevalent at the time, their work laid the foundation for subsequent studies analyzing tumor immune infiltrates including the concept of the Immunoscore(57). The Immunoscore (I) is based on the numeration of two lymphocyte populations (CD3/CD45RO, or CD3/CD8 or CD8/CD45RO) quantified within the CT and IM. These parameters provide a scoring system ranging from Immunoscore 0 (I0), which has low densities of both cell types in both regions; to Immunoscore 4 (I4), having high densities of both cell populations in both regions.(58, 59). Pages et al. in 2018(60) validated the prognostic value of the consensus Immunoscore, which summarizes the density of CD3+ and CD8+ effector T-cells within the tumor and its invasive margin by converting the CD3+ and CD8+ cell densities in these regions to percentiles. The mean of four percentiles (two markers, two regions) was calculated and the tumors classified as low Immunoscore (0-25%), intermediate Immunoscore (25-70%) or high Immunoscore (70-100%). Their study found patients with a high Immunoscore had the lowest risk of recurrence, with significant differences in 5-year disease-free survival between the different Immunoscore groups (HR for high vs low 0·31; intermediate vs low 0·57; high vs intermediate 0·56; p<0·0001). Though the Immunoscore does not specifically characterize immune exclusion, and looking at the invasive margin relative to the tumor core may not be the same as looking the tumor stroma relative to the tumor parenchyma, their work highlights the utility in classifying tumors based on immune contexture, i.e., type, density, and location of immune cells.

Kather et al. (19) studied immune topographies of multiple types of cancers by performing IHC analysis of 965 histological tissue slides from a pan-cancer cohort. They defined three spatial compartments within the tissue specimens: outer invasive margin (0–500 μm outside the tumor invasion front), inner invasive margin (0–500 μm inside the tumor invasion front), and the tumor core (>500 μm inside the invasion front). The authors measured the cell density (number of cells per mm^2^) of a variety of immune cells in each compartment utilizing markers for CD3, CD8, PD1, FOXP3, CD68 and CD163. Their preliminary analysis showed strong correlation between immune cell infiltration into the tumor core and inner invasive margin, and so these two compartments were combined. They then proceeded to define a cutoff value for high versus low cell density of each cell type in the tumor core and outer invasive margin compartments using the median cell density for that cell type (median number of cells per mm^2^ in any tumor type in any compartment). This cut-off value ranged from 5.76 cells/mm^2^ for PD1+ T-lymphocytes to 558.95 cells/mm^2^ for CD163+ macrophages. Using these cutoffs, they defined the three postulated phenotypes of immune topographies of tumors. ‘*Hot*’ or ‘*inflamed*’ tumors were defined as having high cell density inside the tumor regardless of cell density outside of the tumor. ‘*Cold*’ tumors or ‘*immune-desert*’ were defined as having low cell density inside and outside the tumor. Finally, *‘immune excluded’* tumors were defined as having high immune cell density in the outer invasive margin and low density in the core. To validate the clinical utility of their classification system, they analyzed the topography of CD8+ lymphocytes and CD163+ macrophages in colorectal cancer (CRC) primary tumors because these cell types were previously shown to be linked to prognosis (17) and also showed discordant topographies in their pan-cancer cohort. They found a significant association to overall survival when using bivariate immune topographies. With ‘CD8-cold, CD163-cold’ as a reference, the HR was 1.75 for ‘CD8-excluded, CD163 excluded’ (p=0.041) and the HR was 2.71 for ‘CD8-excluded, CD163-hot’ (p=0.025).

Gruosso et al. (61) analyzed formalin-fixed, paraffin embedded (FFPE) samples from a cohort of 38 therapy-naïve triple negative breast cancer patients. They performed IHC to assess spatial distribution and define patterns of CD8+ T cell localization. They defined distinct compartments – the tumor margin, and the tumor core, which was further subdivided into the tumor stroma and tumor epithelium. CD8 + T cell density was quantified in each compartment. Tumors were divided into two groups based on T cell infiltration into the tumor core – corCD8hi (>100 cells/mm2) and corCD8lo (<100 cells/mm2) – and further categorization of corCD8hi tumors was done based on median cell density in the epithelial compartment (epiCD8) of 204.5 cells/mm2. *“Fully inflamed”* (FI) tumors had epiCD8 infiltration above the median (corCD8hi epiCD8hi) while *“Stroma-restricted”* (SR) tumors had epiCD8 infiltration below the median (corCD8hi epiCD8lo). corCD8lo tumors were subdivided based on accumulation of CD8+ T cells at the margin. “*Immune desert*” (ID) tumors had low abundance of CD8+ T cells at the margins (marCD8 <200 cells/mm2) and were classified as corCD8lo marCD8lo while *“Margin-restricted*” (MR) tumors had accumulation of CD8+ T cells at the tumor margins (marCD8 >200 cells/mm2) and were designated as corCD8lo marCD8hi. As an outcome measure, they used GSEA-based metasignatures that best discriminated the subtypes and applied these to an independent external data set for which recurrence-free survival data were available. They were able to show stratification into poor outcome (MR-like), intermediate outcome (SR-like) and good outcome (FI-like), demonstrating the prognostic value of this classification.

Failmezger et al. (62) performed automated morphologic cell classification on H&E sections from 400 melanoma patients available in The Cancer Genome Atlas (TCGA) database to identify lymphocyte, cancer, and stromal cells in each patient’s diagnostic sample. Based on cell spatial mapping provided by automated image analysis, tumor topographs were created where each cell was designated as a “node”, clusters of cancer cells were identified as “supernodes” and edges between cells were drawn based on spatial proximity (<35 μm between them). They then defined measures of network centrality. The *clustering coefficient* measures the degree to of connectivity of the neighborhood surrounding a node with the clustering coefficient of a given node defined as the number of closed triplets divided by the number of all triplets, where a triplet consists of three nodes connected by edges. *Stromal clustering* was defined as the average clustering coefficient of stromal cells within a tumor and *stromal barrier* was calculated by counting the number of stromal cells that a lymphocyte has to cross to reach a cancer cluster. The overall stromal barrier of a sample was calculated as the average of the individual stromal barriers of the lymphocytes in the sample. They then defined four combination groups: low-clustering/low-barrier, low-clustering/high-barrier, high-clustering/high-barrier, and high-clustering/low-barrier. They used overall survival (OS) to assess the prognostic utility of this classification. Their results showed high stromal clustering and barrier were both independently associated with poor 10-year OS, and tumors with high-clustering/high-barrier had significantly worse 10-year OS compared with low clustering/low-barrier tumors. Although their study did not use the term “immune-exclusion”, they defined different measures that in essence reflect an absence of contact between immune cells and cancer cells.

Derks et al. (63) described the spatial distribution of immune cells in different subtypes of gastroesophageal adenocarcinomas. They performed CD8 IHC on 63 archival FFPE surgically resected specimens of untreated gastroesophageal adenocarcinomas to measure the ratio of CD8+ T cell densities at the tumor center (CT) compared to the invasive margin (IM). They then classified tumors as having a ratio of cell densities at CT to IM of >1 or <1. The authors did not use the term “immune exclusion”, but were able to demonstrate profound differences in immune infiltration between gastroesophageal adenocarcinoma subtypes, with Epstein-Barr Virus positive (EBV+) gastroesophageal adenocarcinomas having high CD8+ densities at the tumor center (ratio cell densities TC : IM > 1) whereas most chromosomal instability (CIN) gastroesophageal adenocarcinomas had clustering of CD8+ T cells at the invasive margin (ratio cell densities at TC : IM < 1).

Desbois et al. (54) performed CD8 IHC on 370 archival tissues of patients with ovarian cancer. They developed a digital image analysis algorithm and quantified the total CD8+ T-cell count as well as CD8+ T-cell counts per tumor epithelium and stroma area. These counts were converted into polar coordinates defining two new quantitative metrics: (1) the quantity of CD8+ T cells and (2) the spatial distribution of CD8+ T cells. CD8+ T-cell quantity = [square root ((CD8 tumor)^2^ + (CD8 stroma)^2^)] and CD8+ T-cell spatial distribution = [atan(CD8 stroma/CD8 tumor)]. Next, these two digitally defined quantitative metrics were used to profile the immune phenotype of each tumor using a two-dimensional map with desert tumors having low CD8+ T-cell quantity (R score) and excluded versus infiltrated tumors differing in the spatial distribution of CD8+ T cells (θ score). Their results demonstrated that in the vast majority of tumors, both total CD8+ T-cell quantities and their spatial distribution in the tumor microenvironment are more on a continuum rather than discrete entities. This highlighted the advantage of using their digitally devised two-dimensional quantitative metrics to define tumor immune phenotype.

Hammerl et al. (53) determined spatial immunophenotypes in 4 large cohorts of triple negative breast cancer. In one of the cohorts, they studied CD8+ T cell presence and spatial organization in 236 samples from untreated, primary triple negative breast cancer (TNBC) using IHC. Manual scoring as well as digital image analysis were used to measure CD8 + T cell density at the tumor border and center. For manual scoring, the criteria used were - inflamed: “almost equal frequencies of CD8+ T cells at the border and center”; excluded: “>10 times more CD8+ T cells at the border compared to center”; and ignored: “hardly any CD8+ T cells present at the border and center.” Using digital image analysis, spatial phenotypes were determined according to median CD8+ T cell density at border and center as follows – inflamed: >200 cells/mm^2^ at border and ratio between border and center <10; excluded: >200 cells/mm^2^ at border and ratio between the border and center >10; and ignored: <150 cells/mm^2^ at border and center. They found a significant association of the phenotypes with survival. Tumors with an inflamed phenotype had the best prognosis (10-year OS: 80%), excluded phenotypes intermediate (10-year OS: 60%, HR:1.45, 95% CI: 0.84–3.3), and ignored phenotypes the worst prognosis (10-year OS: 40%; HR:3, 95% CI: 1.5–5.9).

Echarti et al. (52) looked at pre-treatment tissue samples of 280 patients with locally advanced head and neck squamous cell carcinomas (HNSCC). CD8 IHC analysis of FFPE specimens was done, and distribution of CD8+ cytotoxic T lymphocytes (CTLs) was measured in the stromal and intraepithelial compartment of the tumor. CTLs had a median density of 306.5 cells/mm^2^ in the stromal compartment and 235.5 cells/mm^2^ in the intraepithelial compartment. To define the different immunological phenotypes, they determined cut-off values of CTL density. Arbitrarily, the *“immune desert”* group was defined as < 10 CTL/mm^2^ stroma, the *“inflamed”* group as > 1000 CTL/mm^2^ in the epithelium and the *“immune excluded”* group as not meeting either parameter. Kaplan Meier plots for overall survival were used to find a possible difference between the *“immune desert”* and *“immune excluded*” by changing the threshold in steps of 10 CTL. After finding a clear difference (p < 0.010), the authors subsequently repeated this approach comparing *“immune excluded”* and *“inflamed”* groups while using steps of 50 CTLs. The cut off values that were found by this procedure were used in a second round repeating the same procedure, which resulted in cut off values of ≤ 50 cells/mm^2^ in the stromal and > 500 cells/mm^2^ in the epithelial compartment as the best discriminative values regarding overall survival. Thus, cases with less or equal to 50 CTLs/mm^2^ in the stroma were included in the *“immune desert”* group, those with over 500 intraepithelial CTLs/mm^2^ in the *“inflamed”* group. All the cases meeting neither of the two definitions were included in the *“immune excluded”* group. Using median survival as the outcome measure, they showed that patients meeting “immune desert” criteria had an unfavorable prognosis with a median survival of 37.0 months, the “immune excluded” group had an intermediate survival of 61 months and the “inflamed” group tended to have favorable overall survival of 85 months (p = 0.054).

### Gene-based definitions

Some studies have attempted newer techniques such as using gene signatures derived from gene set enrichment analyses to classify tumor immune phenotypes. Mlynska et al. (55) analyzed clinical and transcriptomic data from 489 high-grade serous ovarian carcinoma (HGSOC) patients from The Cancer Genome Atlas (TCGA) database. They selected a set of 40 genes coding for major players in angiogenesis, immune response, and both immune and reactive stroma, based on the evidence of each immune subtype bearing a tumor microenvironment of a distinct nature. The selected genes were used to define gene expression (GE) rules for further patient clustering. A heatmap of expression revealed three distinct biological groups, each representing a specific immune subtype. The inflamed subtype was characterized by high expression of immune response-associated genes and low expression of angiogenesis genes, the excluded subtype had high expression of genes representing the stroma along with low expression of angiogenesis genes, and the desert phenotype showed high expression of angiogenesis genes, with low expression of stromal and immune response genes. Correlating this categorization with clinical data, they observed differences in overall survival among the groups with median OS being 48.7 months, 42.0 months and 40.4 months in the inflamed, desert and excluded subtypes, respectively (p = 0.04).

The previously described study by Desbois et al. (54) went on to develop a gene expression-based molecular classifier using a machine-learning approach to characterize tumor-immune phenotypes. The authors integrated digital pathology and transcriptome analysis and used a random forest regression model to identify genes whose expression could be predicted by the quantity and/or spatial distribution of CD8+ T cells. By performing consensus clustering, they identified 6 clusters with distinct molecular profiles that could be assigned to one of the three defined tumor immune phenotypes. Finally, the authors applied the prediction analysis of microarrays (PAM) approach and built a 157 gene classifier to distinguish the three tumor immune phenotypes. Applying this to a testing set from the ICON7 cohort, they confirmed that the gene expression-based classifier assigned the samples to the appropriate cohort as compared to digital pathology analysis. They also found high concordance between the classifier and manual annotation by a pathologist for immune excluded tumors. Analyzing data from 172 patients enrolled in the chemotherapy control arm of the ICON7 clinical trial with uniform follow-up, they showed that patients with the T-cell excluded phenotype showed significantly shorter progression-free survival (PFS) as compared to patients with the infiltrated or desert phenotypes.

Petitprez et al. (64) studied the gene expression profiles in 608 tumors across subtypes of soft-tissue sarcoma (STS). They analyzed the tumor microenvironment of four independent primary STS cohorts using the microenvironment cell populations (MCP)-counter which is a gene expression-based TME deconvolution tool. Unsupervised clustering of samples in each cohort was done, and the intracohort classifications were aggregated to deduce five pan-cohort sarcoma immune class (SIC) profiles (A-E). SIC A was characterized by the lowest expression of gene signatures related to immune cells, as well as low vasculature, corresponding to the ‘immune desert’ phenotype. SIC E was characterized by the highest expression of genes specific to immune populations such as T cells, CD8+ T cells, natural killer cells, and cytotoxic lymphocytes, corresponding to the ‘immune inflamed’ phenotype. SIC C, ‘vascularized’, was dominated by a high expression of endothelial-cell-related genes. SICs B and D were characterized by heterogeneous but generally immune-low and immune-high profiles, respectively. The authors looked at the clinical outcome of the five SICs and observed that patients with SIC A had the shortest overall survival compared to patients in groups D or E (p = 0.048 and p = 0.025, respectively). Though the authors don’t explicitly define an immune excluded class, their results highlight the likely continuous nature of immune cell infiltration, with their survival analysis suggesting that the intermediate classes (B-D), possibly correspond to the excluded phenotype.

Hammerl et al. (53), in addition to the previously mentioned IHC analysis, also developed a gene expression-based classifier from patient RNA-seq data by identifying genes that were most differentially expressed between patients’ samples as categorized by the IHC analysis. This gene expression classifier was able to correctly assign spatial phenotypes 81% of the time in patients held out as a validation cohort. The gene classifier was then applied to other TNBC cohorts where the immune excluded subtype was shown to be prognostic and predictive of lack of response to PD-1 targeted therapy in the TONIC trial(56). This gene classifier was next applied to a variety of malignancies in TCGA, with the results showing that the immune excluded classification was associated with a worse prognosis and was present in a higher proportion of cancers that do not respond well to immunotherapy, such as prostate and pancreatic cancers.

Xu et al. (65) analyzed immune cell infiltration patterns in breast cancer by employing the genomic and transcriptomic information of 1,198 breast cancer samples from the TCGA-BRCA project and GSE58812 datasets. Using the CIBERSORT computational method, the gene expression information of the TCGA and GEO cohorts was analyzed to obtain a fraction matrix of immune cell infiltration, which estimates the abundances of 22 distinct leukocyte subsets. Using another analytic approach, ESTIMATE, the level of infiltrating immune and stromal cells was predicted by calculating the ESTIMATE, immune, and stromal scores. Finally, single sample gene-set enrichment analysis (ssGSEA) was conducted based on the expression level of 29 immunity-associated signatures. The samples were then clustered into 3 discrete subgroups according to similarities exhibited in the immune cell infiltration profiles. Thus, three different immune cell infiltration patterns were finally identified by using an unsupervised clustering. Cluster A was characterized by infiltration of quiescent and innate immune cells along with stromal activation and was considered to exhibit an immune-excluded phenotype; Cluster B was characterized by a weakened immune cell infiltration and was identified as having an immune-desert phenotype; and Cluster C was characterized by an elevated inflammation response and was recognized as showing an immune-inflamed phenotype. Kaplan-Meier survival analysis of the three distinct patterns indicated that Cluster A exhibited a prominent advantage of median survival time, whereas Cluster B presented with the worst prognosis (p = 0.021).

## Need for a consistent practical definition of immune exclusion

With the increasing role of immunotherapy in treatment of cancers and investigations into immune resistance ongoing to understand which patients are most likely to benefit, it is a pressing question to understand how cancer immune phenotypes correlate to outcome and response to immunotherapy. The study by Hammerl et al. (53) specifically looked at response to immunotherapy with the different phenotypes and established higher prevalence of the excluded phenotype in non-responders, though it is limited by a small dataset. Other studies looking at response to immunotherapy have also established better response rates in the inflamed phenotype, though they do not make the distinction between excluded and cold tumors. Given the association between immune exclusion and resistance to existing immunotherapies, there is potential to target the underlying mechanism (or mechanisms) of immune exclusion to improve patient outcomes. Data also suggest that the biology underpinning immune exclusion does not mirror the other phenotypes and therefore requires new tools, insights, and methods to dissect it and translate it into actionable science.

The existing literature lacks in consistency when it comes to practically defining immune exclusion, which limits the interpretation and cross-study comparison of the results. To better understand the relative impact of the immune excluded phenotype on prognosis and response to treatment for different cancer types, and to have an operational definition to apply in clinical trials and ultimately clinical practice, an objective definition of immune exclusion is necessary.

The definitions that have been used in the literature, while they have been very valuable in raising awareness and identifying the biologic and prognostic associations of immune exclusion, have common limitations that can be addressed in future studies. For one, they attempt to place each tumor into a distinct phenotypic category, at times using somewhat arbitrary cut-offs, an attempt that is not well suited for describing immune phenotypes which are notably complex and comprised of continuous variables. The work of Desbois et al. (54) highlights that the number and distribution of immune cells in tumors is a wide-ranging spectrum, an attribute that can be lost with overly simplified definitions. Another limitation is that they often do not account for tumor heterogeneity, i.e., the variation in composition and spatial distribution of components of the TME that occurs within an individual patient’s tumor (18, 66, 67). And of course, differences in the presentation of immune exclusion within different tumor types may necessitate different approaches and criteria based on cancer indication.

## A new approach to defining immune exclusion

The phenomenon of immune exclusion has been suggested to be a continuum (22, 54). To better evaluate this, we propose that investigators should consistently report the degree of immune infiltration, particularly CD8+ lymphocytes, in the parenchyma of the tumor and the surrounding stroma as density of immune cells and the ratios between the compartments. This will aid in cross-study comparisons to further refine the definition. Majority of the existing data looking at immune cell infiltrates and their correlation with outcomes has focused on CD8+ T-cells, hence their importance in this regard is well established. As the study by Xu et al. (65) illustrated, the activation status of infiltrating CD8+ T cells and nature of other infiltrating or excluded immune cell subsets could be an important parameter to include when defining immune exclusion. It is increasingly recognized that gamma-delta T cells, NK cells and plasma cells have significant contribution to the overall anti-tumor response, therefore delineating the spatial distribution of these cell types should also be considered (68). NK cells in particular can serve as powerful effectors of the innate immune response by killing transformed cells and have been shown to play an important role in suppressing metastasis (69). However, their activity against established solid tumors is limited by their inability to infiltrate the tumor core, with studies showing localizing of NK cells in the tumor stroma in certain cancers reflective of “immune excluded” phenotype, though they are not defined or objectively measured as such.(70, 71) As more data emerges and clarifies the role of other immune cell types and subtypes in cancer, their degree of infiltration may perhaps also become relevant. The different immune cell phenotypes and subsets may be reported as absolute cell densities, percentage of total immune cells or normalized to all cells analyzed. At this moment, however, more data is needed to establish the most useful measure and we are not in a position to define that, especially as it relates to the different cancer histologies. Cut-offs to distinguish categorical phenotypes could be tied to clinically meaningful outcome measures, as demonstrated by the work of Echarti et al. (52) where categorical cut-off values were selected to best discriminate overall survival differences. Another potential differentiator could be response to immunotherapy as a clinically relevant classification (**Table 1**).

**Table 1:** Recommendation for evaluation and reporting Immune Exclusion in cancer samples.

	Recommendation	Notes
Tissue sample	Surgical resection specimen or core needle biopsy with multiple cores	Locations of metastasis may impact the immune phenotype
Analysis method	Immunohistochemistry or immunofluorencence with a marker to differentiate stroma from tumor parenchyma	Gene signatures should be derived from a sufficiently large dataset of tumors with spatial characterization
Immune cells analyzed	CD8+ T lymphocytes	Evaluating T cell activation/exhaustion markers and other immune cell subsets add additional information of the TME contexture
Compartments analyzed	Tumor parenchyma	
	Tumor Stroma	
Measured value	Cell densities (cells/mm^3^)	
	Ratio of parenchyma/stroma	
Cut-off for definition	Based on clinical outcome variables as available	These include overall survival, progression-free or disease-free survival, and response to therapy
Tumor heterogeneity	Evaluate multiple areas of tumor	Report degree of heterogeneity

To address issues of tumor heterogeneity, we propose that efforts should be made to assess as many areas of a given tumor sample as is practically possible. This becomes a limitation when dealing with core needle biopsies, that can be addressed, in part, by obtaining multiple cores, when feasible. While averaging the immune infiltrate density values would be more reflective of a sample than any one evaluated portion, it may be most valuable to report the degree of heterogeneity, as this coefficient may have its own biologic and prognostic implications (72–74). Towards this end, approaches could be used that evaluate immune cell density in the tumor center as compared to the invasive margin, or a grid or patch like approach could be adopted to give a fuller evaluation of differences in immune cell infiltration across a tumor sample (**Table 1**).

Achieving clinically relevant definitions of immune phenotypes that are not reliant on IHC staining and manual counting would allow large association studies to be performed more quickly and with less cost using existing datasets. Artificial intelligence (AI) and deep learning have the potential to quickly evaluate large quantities of slides by automating the simpler, repetitive and time-consuming tasks while allowing the pathologists to spend additional time on high level decision making. AI-based approaches, particularly CD8 IHC with quantification of CD8+ T cells relative to the tumor parenchyma and tumor-associated stroma, allow extraction of multiple subvisual morphometric features, potentially enabling the evaluation of immune exclusion from diagnostic H&E-stained slides using morphology-based cell and tissue classification (75). Routine H&E slides offer the most practical method, with the potential to be integrated into routine clinical workflows. As Failmezger et al (62) showed, automated image analysis from H&Es also allows for more complex and sophisticated measures to be defined, though this methodology is yet to be validated against the gold standard IHC. AI-based approaches in IHC could offer more specific and sophisticated analyses, though it would likely be more challenging to develop.

Gene signatures as reviewed above, also appear to hold great promise. There are certain limitations, specifically, bulk RNA sequencing, as it is confounded by variable levels of tumor cell purity. Within those limitations, consistently across studies, there appears to be an increased expression of stromal genes in the immune excluded phenotype, likely secondary to a higher proportion of stroma within the tumor, and increased expression of immune/inflammation genes in the immune inflamed phenotype due to the presence of higher levels of immune cells. Given the variability in the approaches used so far, further validation and consensus on these signatures within individual histologic types and between different types of cacer is needed. Most gene-based studies have used an unsupervised clustering approach. As the understanding of molecular signatures of different phenotypes improves, a practical approach to gene-based definition might include tying the expression of relevant genes and signatures in each phenotype with meaningful clinical outcomes to define cut-offs, in a “semi-supervised” clustering approach (76).

It is unclear if one definition of immune exclusion will apply across all tumor types, but there would be value in achieving one definition, understanding that some histologies will have a higher proportion of immune excluded cancers than others, and the clinical implications of the phenotype may vary between histologies. As a natural extension, a consensus definition of immune exclusion would lead to more concrete definitions of immune inflamed and immune desert phenotypes as well. A unified definition will also help allow better determination of the underlying mechanisms of immune exclusion that span cancer types and, ultimately, aid in the development of treatments to target these mechanisms.

## Conclusion and recommendations

As the understanding of the immune landscape of cancer has improved, the concept of immune phenotypes, as it relates to patient outcomes and response to immunotherapy, has become increasingly relevant. This has led to further investigations into the phenomenon of immune exclusion, where effector immune cells are present at the site of the tumor but unable to effectively engage tumor cells. However, as a result of those investigations, which have been carried out by multiple groups to assess immune exclusion from multiple perspectives, there now exist varied and inconsistent practical definitions of this concept.

In this review, we have looked at the various studies and diverse definitions of immune exclusion and identified a key issue of establishing clinically relevant cut-offs within the spectrum of immune cell infiltration. We also bring forth tumor heterogeneity as a major variable to factor in when evaluating immune exclusion.

Moving forward, we propose that future studies focused on the spatial phenotypes of cancer consistently report the degree of immune infiltration, particularly CD8+ lymphocytes, in the parenchyma of the tumor and the surrounding stroma as density of immune cells and the ratios between the compartments. We further propose that efforts should be made to assess as many separate areas of a given tumor sample as is practical. This data with matched clinically meaningful endpoints, such as survival and response to therapies, will allow the development of histology-specific cutoffs that can be then be used in clinical trials to develop therapeutics to address the mechanism driving exclusion of T cells.

We offer that taking these factors into account when analyzing immune exclusion will help in achieving a consistent, practically feasible, and clinically pertinent definition of immune exclusion which could be applicable across a wide range of cancer histologies, tissue analysis methods and study designs. This, in turn, will allow for an improved understanding of the concept, aid in understanding the potential mechanisms involved, and further progress to uncover potential therapeutic targets to improve patient responses.

## Author contributions

AT and GC prepared and drafting of the manuscript. GC and LA planned the concept and scope of the review. AT, TO, LD, LA, AI, WF, and GC all substantially edited the review. All authors contributed to the article and approved the submitted version.
