# Supplementary material for: Towards a consensus definition of immune exclusion in cancer
Source: Front Immunol. 2023 Mar 22;14:1084887. doi: 10.3389/fimmu.2023.1084887 (PMC10073666; doi:10.3389/fimmu.2023.1084887)
Supplement: Supplementary file 1 [file Table_1.docx]

Supplementary Material

# Supplementary Table: Previously applied definitions of immune exclusion by various studies

| Study | Type(s) of Cancer | Method for analyzing immune cells | Immune cells analyzed | Criteria used for classification of phenotypes | Definition of immune exclusion or equivalent phenotype(s) |
| --- | --- | --- | --- | --- | --- |
| Kather et al 2018 | Multiple | IHC | CD3+ T-lymphocytes, CD8+ T-lymphocytes, PD1+ T-lymphocytes, FOXP3+ T-lymphocytes, and CD68+ and CD163+ monocytes/macrophages | Median cell density for each cell type (median number of cells per mm^2^ in any tumor type in any compartment) | High immune cell density in the outer invasive margin and low density in the core |
| Gruosso et al 2019 | Triple negative breast cancer | IHC | CD8+ T-lymphocytes | Tumor core - 100 cells/mm2  Epithelial compartment - Median cell density (204.5 cells/mm2)  Tumor margin – 200 cells/mm2 | *“Stroma-restricted”* (SR) - epiCD8 infiltration below the median (corCD8hi epiCD8lo)  “Margin-restricted” (MR) - accumulation of CD8+ T cells at the tumor margins (marCD8 >200 cells/mm2), designated as corCD8lo marCD8hi |
| Failmezger et al 2019 | Melanoma | Automated image analysis using computati-onal pathology | All lymphocytes | Clustering coefficient = Number of closed triplets/Number of all triplets, where a triplet consists of three nodes with edges connecting all of them  *Stromal clustering* = average clustering coefficient of stromal cells within a tumor  *Stromal barrier* = number of stromal cells that a lymphocyte has to cross to reach a cancer cluster | High-clustering/high-barrier |
| Echarti et al 2019 | Locally advanced HNSCC | IHC | CD8+ T-lymphocytes and FoxP3+ T-lymphocytes | < 50 cells mm^−2^ in the stromal and > 500 cells mm^−2^ in the epithelial compartment | *“Immune desert”* - less or equal to 50 CTLs mm^−2^ in the stroma.  *“Inflamed”* - over 500 intraepithelial CTLs mm^−2^.  All the cases meeting neither of the two definitions were included in the *“immune excluded”* group |
| Derks et al 2020 | Gastroesophageal adenocarcinoma | IHC | CD8+ T-lymphocytes | Ratio of cell densities at tumor center to invasive margin | Ratio of cell density at tumor center to invasive margin <1 |
| Desbois et al 2020 | Epithelial ovarian cancer | IHC | CD8+ T-lymphocytes | Two-dimensional map using digitally defined quantitative metrics, namely, the quantity and the spatial distribution of CD8+ T-lymphocytes | Tumors with high total quantity of CD8+ T-lymphocytes, with a larger proportion of the cells in the stroma. However, they demonstrated a continuum of total CD8+ T-cell quantities and their spatial distribution without discrete cut-offs |
| Hammerl et al 2021 | TNBC | IHC, Multiplexed IF | CD8+ T-lymphocytes, CD4+ T-lymphocytes, CD56+ NK cells, CD20+ B-lymphocytes and CD68+ macrophages | Ratio of cell densities between border and center | >10 times more CD8+ T cells at the border compared to center |
